# A Systematic Evaluation of Protein Phase Separation Predictors across Diverse Protein Landscapes

**DOI:** 10.34133/csbj.0115

**Published:** 2026-06-01

**Authors:** Kate E. Gilroy, John N. Barr

**Affiliations:** ^1^ School of Molecular and Cellular Biology, Faculty of Biological Sciences, University of Leeds, Leeds, UK.; ^2^ Astbury Centre for Structural Molecular Biology, University of Leeds, Leeds, UK.

## Abstract

**Background:** Liquid–liquid phase separation (LLPS) plays a central role in cellular regulation, with its dysregulation linked to numerous diseases. LLPS is also increasingly implicated in various biological contexts, such as virus replication. These findings have driven the development of numerous computational predictors to screen and identify phase-separating proteins from sequence and/or structural models. Despite the need for these tools, their performance across diverse biological contexts remains incompletely understood, complicating tool selection and result interpretation. **Results:** We performed a systematic comparative analysis of 9 LLPS prediction algorithms using multiple curated datasets comprising both LLPS-positive (LLPS+) and LLPS-negative (LLPS−) proteins. The datasets span multiple biologically relevant scenarios, including intrinsically disordered proteins, folded proteins, proteins with LLPS-abolishing variants, benchmark datasets, and viral proteins. We observed substantial variability in predictive performance across algorithms when assessing proteins of different structural classes such as LLPS+ and LLPS− folded proteins, LLPS-abolishing mutations, and viral proteins. **Conclusions:** These results demonstrate that LLPS predictor performance is strongly context dependent, leading to different predictors being optimal for different biological questions. For overall protein assessment, DeePhase and MolPhase provided the most consistently accurate predictions, being the least impacted by structural bias. For assessing the impact of small mutations on LLPS propensity, PSPHunter, a built-for-purpose algorithm, reliably predicts mutation impacts, with structure-informed algorithms PSPire and PICNIC also providing strong insight. Across all evaluated datasets, the findings highlight the need for well-benchmarked training and testing data that encompass a broad and diverse range of protein classes.

## Background

Biomolecular condensates are cellular organelles or structures that lack a delimiting membrane but still function in concentrating and segregating cellular components [1–4]. In recent years, the regulation of such structures has been identified as often occurring via the process of liquid–liquid phase separation (LLPS), causing the demixing of macromolecular components in a solution [5]. This finding has led to greater understanding of numerous essential cellular structures and processes, including P-bodies and stress granules [2,6], and its dysregulation has been linked to numerous disease outcomes [7–11]. While there have been great strides made in the ability to experimentally identify phase-separating proteins (PSPs), it remains a laborious task often requiring extensive *in vitro* and *in cellulo* investigations [12]. Due to the increased recognition of the importance of LLPS in the regulation and dysregulation of biological systems, more high-throughput screening methods that do not require these time-consuming experiments are essential to identify potential PSPs.

Some of the earliest computational predictors used for LLPS date back to 2014 [13] but were not initially designed for specific prediction purposes. PLAAC predicts prion-like domains [13], R+Y assesses the arginine and tyrosine content of a protein [14], and PScore calculates planar pi-interaction frequency [15]. After their production, however, researchers recognized their utility as LLPS predictors, since many of the characteristics that these algorithms assess are identifiers of PSPs [16–22]. Despite their importance early on, these methods have now become outdated by a second generation of designed-for-purpose LLPS prediction algorithms, many of which incorporate machine learning models, learning patterns from a wide array of features including amino acid composition and intrinsic disorder, rather than the single-feature approach [23]. These algorithms also have access to a larger range of datasets for testing and training, increasing their abilities.

The majority of predictors look at “sequence-based” features, focusing on the biochemical properties of each amino acid within the primary protein sequence as it has been proposed that this is where the LLPS capacity of a protein is defined [24,25]. However, it has been argued that sequence features alone are insufficient for accurate prediction, and structural aspects also need to be considered [26]. Adding structural features to predictors, however, had been limited due to the lack of high-quality protein structures. However, since the wide availability of AlphaFold [27,28] to the public, this has changed, with near-experimental-quality structures being produced and deposited in the AlphaFold Protein Structure Database [29,30]. This has led to the recent development of a potential “third generation” of LLPS predictors that not only assess sequences but also consider structure-based characteristics such as structured superficial regions and secondary arrangements. Predictors employing this third-generation approach include PICNIC [31] and PSPire [26], claiming to identify PSPs with higher accuracy than both first- and second-generation algorithms. Interestingly, both also assert the ability to identify PSPs regardless of intrinsically disordered regions (IDRs), one of the most well-established features driving LLPS [32]. Due to the large contribution of IDRs to LLPS, most predictors consider them as a main indicator of phase separation potential; however, a growing number of proteins with no, or limited, IDRs have been found to undergo phase separation [26,33]. Additionally, the mere presence of IDRs is not enough for many proteins to phase separate under physiological conditions [34], and thus, this bias in predictors can lead to both false positive and false negative results. This poses the question of whether third-generation, structure-informed predictors that look beyond IDRs will provide the most accurate results or if structure is not required for accurate LLPS predictions.

Another approach to the computational understanding of LLPS also exists in the form of molecular dynamics simulations and coarse-grained models. These models allow for the prediction and dissection of protein and biomolecular condensate behavior including protein interactions, saturation concentrations, and production of phase diagrams [35–40]. While invaluable within LLPS research, molecular-dynamics-based methods are often computationally more intensive and require careful configuration to gain meaningful results, making them inaccessible to many researchers with a nonbiophysical background, and thus will not be assessed in this paper.

Despite these recent advances in, and new directions approached by, LLPS prediction algorithms, there have been few unbiased comparative assessments of these different predictive approaches. Liao *et al.* [41] performed a comparative analysis of numerous first- and second-generation algorithms, confirming the better predictive performance of machine-learning-based approaches. However, in the few years since their study, the third generation of structure-based, IDR-unbiased approaches has been released, requiring assessment and comparative analysis of predictor performance using structural models. Additionally, the growing number of second-generation algorithms are constantly changing model architecture and protein features assessed to find the optimal model build for prediction, requiring an unbiased comparison between second-generation predictor performance. There are also additional unaddressed concerns regarding the usefulness of these algorithms, beyond some limited scenarios, that have not yet been investigated. These include biases in the training and testing datasets used to develop these predictors, the ability to detect changes in LLPS propensity caused by small mutations, the ability to identify regions responsible for driving phase separation, and the ability to accurately predict phase separation for underrepresented organisms with limited homologs, such as viruses [42–44]. As understanding of the diverse roles of LLPS in a variety of biological systems grows, it is important that prediction algorithms develop alongside. Therefore, assessing current predictors for their fitness in such complex and diverse scenarios, as well as identifying areas necessitating development, is crucial.

To address these issues, here, we performed a comprehensive comparative analysis of 9 second- and third-generation LLPS predictors: MolPhase [45], DeePhase [46], FuzDrop [47–49], PSPHunter [50], LLPhyScore [24], PSAP [51], PSPire [26], Phaseek [52], and PICNIC [31]. Each predictor was compared across a variety of datasets including a “Standard” testing dataset, a selection of LLPS-abolishing mutants, sequences of varying structural classification, and a set of LLPS-positive (LLPS+) and LLPS-negative (LLPS−) viral protein sequences. Additionally, the effectiveness of third-generation predictors utilizing structural protein models was compared to that of the sequence-based second-generation predictors. The performance of all algorithms was then analyzed for overall prediction power in addition to accuracy in specialist scenarios to aid researchers in identifying the best-performing algorithm for their applications. Additionally, this provides an overview of the weaknesses and strengths in the current LLPS prediction research environment and ways in which the field can move forward.

## Methods

### Selecting phase separation predictors for evaluation

A targeted literature search was undertaken to identify second-generation phase separation predictors. This included those employing machine or deep learning algorithms and/or those utilizing multiple engineered features and large datasets specifically designed for phase separation prediction. The search was then extended to include predictors that assess structural protein models in addition to sequence features—the potential third-generation algorithms. Focus was placed on algorithms that provided easy-to-implement interfaces or instructions and those that are not computationally intense on the user end, so the algorithms are accessible to as many potential users as possible. It must be noted that, despite efforts to make the literature review as comprehensive as possible, some algorithms were potentially missed. This returned 11 predictors, 9 second-generation—MolPhase [45], DeePhase [46], FuzDrop [47–49], PSPHunter [50], Seq2Phase [53], PSPredictor [54], LLPhyScore [24], PSAP [51], and Phaseek [52]—and 2 third-generation—PSPire [26] and PICNIC [31]. Of these predictors, 2 were eliminated: PSPredictor and Seq2Phase. PSPredictor was excluded as, at the time of this analysis, the web-based interface was unavailable, and no code was made publicly available to run locally, making it inaccessible. The other excluded predictor, Seq2Phase, provides the code required; however, it involves the user training the Seq2Phase model locally. This requires a large amount of computing power to be completed quickly and successfully, making it inaccessible to many potential users. Thus, the focus of this study was placed on algorithms that are most accessible for researchers of all backgrounds. Key details of the 9 selected algorithms can be seen in Table 1. DeePhase, MolPhase, FuzDrop, and PSPHunter were used via their browser-based interface; Phaseek was utilized via a Google Colab notebook; and the remaining algorithms were utilized via publicly available code linked in the relevant manuscripts.

**Table 1.** Key details of the chosen LLPS predictors

Predictor	Model construction	Key features analyzed	Output	Availability		Refs.
				Code?	Web-based interface?	
MolPhase	Machine learning XGBoost classifier	Sequence length, % IDRs and LCRs, PLDs, type II polyproline helices, pi interactions, Shannon entropy, charge–charge interactions, hydrophobicity, FCR, NCPR, kappa and omega, protein-level amino acid composition	Overall protein prediction score Protein-level scores of key features Residue-level scores of key features	No	Yes	[30]
DeePhase	Machine learning Neural-network-based word2vec with random forest classifiers	Sequence length, hydrophobicity, Shannon entropy, % IDRs and LCRs, LCR amino acid composition, protein-level amino acid composition	Overall protein prediction score Graph representing residue-level prediction scores	Yes	Yes	[31]
FuzDrop	Supervised statistical predictor Binary logistic model	Conformational entropy changes, hydrophobicity, intrinsic disorder	Overall protein prediction score Residue-level score or droplet-promoting probability Regions categorized as droplet promoting or as containing context-dependent interactions	No	Yes	[32–34]
PSPHunter	Machine learning Random forest classifier using word2vec with PSSM and HMM	Sequence length, amino acid composition, functional site annotations, PTMs, IDRs, RNA + DNA binding regions, evolutionary conservation, network characteristics	Overall protein prediction score Residue-level prediction scores and driving regions Heatmap of the overall score changes caused by missense mutations at each residue	Yes	Yes	[35]
LLPhyScore	Machine learning 3-layer neural network-style model	Protein–water interactions, protein–carbon interactions, long-range hydrogen bonds, long-range pi–pi interactions, long-range disorder, K-beta similarity, short-range disorder, short-range electrostatic interactions	Protein-level scores for the 8 key protein features 8-feature sum representing final prediction score	Yes	No	[16]
PSAP	Machine learning Random forest classifier	Amino acid composition, % IDRs and LCRs	Protein-level relative score with intrabatch ranking	Yes	No	[36]
PSPire	Machine learning XGBoost classifier	Structured superficial (SSUP) regions, sticker and spacer regions, PTMs, secondary structure	Overall protein prediction score SSUP regions identified Positive and negative sticker regions identified	Yes	No	[17]
PICNIC	Machine learning Gradient boosting machine classifier (optional GO module)	IDRs + LCRs, secondary structure, short- and long-range amino acid composition	Overall protein prediction score	Yes	No	[22]
Phaseek	Machine learning GPT encoder-based protein graph generator and XGBoost bottleneck	IDRs, hydrophobicity, amino acid composition, type II polyproline helices, charge patterning	Overall protein prediction score Residue-level prediction scores	Yes	Yes	[37]

LLPS, liquid–liquid phase separation; IDRs, intrinsically disordered regions; LCRs, low-complexity regions; NCPR, net charge per residue; GO, Gene Ontology; FCR, fraction of charged residues; PSSM, position-specific scoring matrix; HMM, hidden Markov model; PTMs, posttranslational modifications; GPT, generative pre-trained transformer

### Dataset construction

To avoid any overlap of sequences used in this analysis to those used to train the predictors, all training sequences from each predictor were collected from the Methods or Supplementary Materials section of each manuscript. For each dataset described below, proteins that had more than 40% sequence identity with any training sequence were identified and removed via CD-HIT [55].

#### “Standard” dataset

The standard dataset was created to represent the commonly used process of generating positive and negative datasets for both training and testing most prediction algorithms. For the LLPS+ dataset, driver-exclusive, experimentally validated PSP sequences were collected from the database MLOsMetaDB [56]. For the LLPS− dataset, the Protein Sequence Culling Server (PISCES) [57] was used to collect protein sequences with high-resolution x-ray structures containing no chain breaks or breaks due to disorder. These proteins are commonly used as negative sequences as those with low- to no-disorder and well-established structures are unlikely to undergo phase separation. Any proteins containing noncanonical amino acids, shorter than 60 amino acids, or with over 40% sequence identity between positive and negative classes were removed. Biopython SeqIO [58] was then utilized to randomly select 100 positive and 100 negative sequences, forming the “Standard” dataset.

#### “Disordered vs. Folded” dataset

Despite the evidence that proteins with an ordered structure are less likely to phase separate and those with disorder are more likely to, this is not always true [26,33]. To assess if the predictors are affected by this bias, LLPS+ and LLPS− classes were further categorized as “Disordered” or “Folded”. The LLPS+ sequences were generated as per the “Standard” dataset; however, prior to condensing to 100 sequences, disorder was assessed via Metapredict [59]. Sequences with Metapredict scores <0.5 were considered “Folded”, and those with scores ≥0.5 were considered “Disordered”. SeqIO was then utilized to collect 100 randomized sequences of each.

The LLPS− dataset outlined in the “Standard” dataset was utilized as the “Folded” category with another 100 sequences being randomly selected by SeqIO. For the LLPS− “Disordered” proteins, the database DisProt [60] was used. All nonobsolete sequences were collected, and those marked as “condensates-related proteins” by DisProt were removed. Additionally, any sequences with >40% sequence similarity to any folded or disordered proteins in this dataset were removed by CD-HIT, as well as those with noncanonical amino acids or sequences shorter than 60 residues; 100 sequences were chosen at random by SeqIO.

To account for changes in LLPS prediction power due to modest changes in the Metapredict threshold used to categorize the LLPS+ disordered and folded datasets, a sensitivity analysis was undertaken. From the 100 randomly chosen LLPS+ disordered sequences, any proteins with a score <0.7 were removed and the remaining sequences formed the “LLPS+ Disordered >0.7” dataset. Similarly, from the 100 random sequences chosen as LLPS+ folded, any proteins with a score >0.3 were removed and the remaining sequences formed the “LLPS+ Folded <0.3” dataset.

#### “Benchmark” dataset

It is currently hypothesized that almost any protein, regardless of structural status, has the potential to undergo phase separation under specific, although often nonphysiological, conditions [61,62]. This makes it difficult to produce a “true negative” dataset for predictor training and testing as there are almost certainly false negatives included. To challenge this, Pintado-Grima *et al.* [63] attempted to produce high-confidence “Benchmark” LLPS+ and LLPS− datasets utilizing strict filters for curation. For the LLPS+ dataset, proteins identified as “driver-exclusive” were selected, and the full LLPS− dataset was used. Any proteins containing noncanonical amino acids, shorter than 60 amino acids, or with over 40% sequence identity between positive and negative classes were removed. SeqIO was then utilized to randomly select 100 positive and 100 negative sequences.

#### “Mutations” dataset

It has been well documented that in many proteins, small and/or single-amino-acid mutations can substantially alter or abolish the ability of a protein to undergo phase separation [64–66]. It is therefore important to assess the capability of predictors to detect these small but impactful changes and reflect this in their scores. LLPSDB v2.0 [67] allows filtration of PSPs to identify those in which the wild-type (WT) sequence undergoes LLPS, but specific mutations have been experimentally validated to disrupt this ability. Following culling of training proteins, 12 WT sequences remained. Unique mutation sequences from LLPSDB v2.0 for the corresponding WT sequences were then collated. In this dataset, the LLPS+ proteins are the WT sequences and the LLPS− proteins are the mutant sequences.

#### “Virus” dataset

To assess if the predictors can accurately assess the LLPS ability of proteins from an underrepresented class, viral proteins were collected. LLPS+ viral sequences were collected from BAV-LLPS-DB [68] and unsuitable proteins were filtered out as with previous datasets, resulting in 30 LLPS+ viral sequences.

For LLPS−, viral sequences with high-resolution x-ray structures were collected from the Protein Data Bank (PDB) and filtered as with previous datasets; 30 random sequences were then selected by SeqIO.

**Fig. 1.**
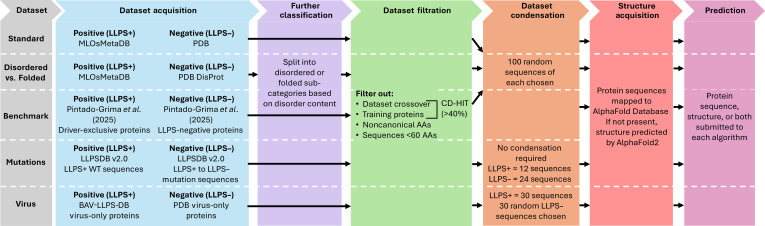
Workflow of dataset construction. Overview of the acquisition, filtration, and analysis steps taken for each dataset utilized in this study.

As PSPire and PICNIC require structural files for their predictions, with a preference for AlphaFold structures, the corresponding structures were collected from the AlphaFold database [29,30]. However, where there was no structure available, AlphaFold2 [27,28] was used to produce batch predictions. An overview of the dataset construction workflow can be seen in Fig. 1, and the sequences used in each dataset are outlined in Tables S1 to S12.

### Data analysis and evaluation parameters

To assess the overall performance and accuracy of the predictors, receiver operating characteristic (ROC) curves and precision–recall curves (PRCs) were constructed. ROC curves are generated by varying the decision threshold across the full range of potential scores (0.0 to 1.0). The endpoint of a true positive rate and a false negative rate of 1 is a trivial endpoint where all proteins are classified as LLPS+ and does not represent a meaningful value. PRCs are similarly generated but do not contain fixed endpoints. The area under the curve for each was then calculated (area under the ROC curve [AUROC] and area under the PRC [AUPRC], respectively). Curve construction and area under analysis was completed utilizing Python package sklearn [69].

$$\text{Precision}=\frac{\mathrm{TP}}{\left(\mathrm{TP}+\mathrm{FP}\right)}$$ (1)

$$\begin{array}{c} \text{True positive rate}\;\left(\mathrm{TPR},\;\text{recall}\right)=\frac{\mathrm{TP}}{\left(\mathrm{TP}+\mathrm{FN}\right)} \end{array}$$ (2)

$$\text{False positive rate}\;\left(\mathrm{FPR}\right)=\frac{\mathrm{FP}}{\left(\mathrm{FP}+\mathrm{TN}\right)}$$ (3)

$$\text{AUPRC}=\sum_{n}\left(R_{n}-R_{n-1}\right)P_{n}$$ (4)

$$\text{AUROC}=\sum_{i=1}^{n-1}\left({\mathrm{FPR}}_{i+1}-{\mathrm{FPR}}_{i}\right)\times \frac{{\mathrm{TPR}}_{i}+{\mathrm{TPR}}_{i}}{2}$$ (5)

In the above equations, *TP* is true positive, *FP* is false positive, *FN* is false negative, *TN* is true negative, *R* is recall, *P* is precision, and *n* and *i* are indices denoting successive operating points obtained by varying the classification threshold to build the curves.

For all datasets except “Disordered vs. Folded”, AUROC and AUPRC were calculated from the LLPS+ and LLPS− sequences within the same dataset. For “Disordered vs. Folded”, AUROC and AUPRC were calculated for “Folded-Only” sequences (“LLPS+ Folded vs. LLPS− Folded”), “Disordered-Only” sequences (“LLPS+ Disordered vs. LLPS− Disordered”), and “Mixed” sequences (“LLPS+ Folded & Disordered vs. LLPS− Folded & Disordered”). For the disorder sensitivity analysis, the same AUROC analysis was performed but with changing either “LLPS+ Disordered” to “LLPS+ Disordered >0.7” or “LLPS+ Folded” to “LLPS+ Folded <0.3”. For all algorithms, only the protein-level score was used for comparison.

AUROC and AUPRC confidence intervals (CIs) were obtained using a stratified nonparametric bootstrap. LLPS+ and LLPS− proteins were resampled with replacement within each class to preserve the original class balance. For each predictor, 5,000 bootstrap datasets were generated and AUROC/AUPRC were recomputed using sklearn [69]. The 95% CI was taken as the 2.5th to 97.5th percentile of the bootstrap distribution. The same procedure was repeated for the “Folded-Only”, “Disordered-Only”, and “Mixed” subsets of the “Disordered vs. Folded” dataset.

To calculate fold change in prediction score with the “Mutations” dataset, the following equation was used:

$$\begin{array}{c} \text{Fold change}=\log_{2}\left(\frac{\text{Mutation prediction score}}{\mathrm{WT}\;\text{prediction score}}\right) \end{array}$$ (6)

For generating a final “rank”, each algorithm was scored from 1 to 9 on performance in each individual dataset with the highest AUROC score being rank 1 and the lowest AUROC score being rank 9. The mean rank for each algorithm across all datasets was then calculated and returned as the overall rank.

### Gene Ontology analysis

The UniProt accessions of the “Standard”, “Disordered vs. Folded”, and “Benchmark” datasets before culling to 100 random sequences were analyzed via Database for Annotation, Visualization, and Integrated Discovery (DAVID) [70,71]. Due to the mixed taxonomy of the datasets, only proteins with annotations in *Homo sapiens* were used. Gene Ontology (GO) terms were collected from the following databases: GOTERMS_MF_DIRECT, GOTERMS_CC_DIRECT, and UP_SEQ_FEATURE. The threshold for the number of hits per term was set as >3 hits, the *P* value was set as ≤0.05, and fold enrichment was set as >2.

### Protein feature analysis

The Biopython Python package was utilized to calculate the key sequence features of the “Standard”, “Disordered vs. Folded”, “Benchmark”, and “Virus” datasets. The features calculated included length (number of amino acids in sequence), hydropathy (using the Kyte–Doolittle hydropathy scale), aromatic fraction (proportion of tyrosine, phenylalanine, and tryptophan in sequence), low-complexity region (LCR) fraction (using Shannon entropy), and net charge per residue (NCPR; the net charge of the sequence divided by length). Due to the nonevenly distributed nature of the datasets, a Mann–Whitney *U* test was used to determine significance between and within datasets.

## Results and Discussion

### Performance of prediction algorithms on a standard LLPS testing dataset

In the production, training, and testing of LLPS algorithms, there are common methods in which datasets are curated. For LLPS+ proteins, curation has been made simpler via the recent advances in identifying these proteins experimentally and the subsequent development of databases to collate and categorize them [56,72–75]. For LLPS− datasets, it is substantially more complex. Due to the nature of LLPS, it is nearly impossible to identify a protein as a “true negative” as many proteins undergo LLPS only under specific conditions [12,20], of which it would not be feasible to test every protein against. Instead, negative datasets are often built based on the idea that proteins with well-documented nonflexible structures are much less likely to undergo phase separation than those with a flexible, disordered nature. Therefore, taking a collection of proteins with high-resolution x-ray structures from databases like the PDB [76] is the most common method of building an LLPS− dataset. This “standard” approach is no longer considered correct as the understanding of proteins that phase separate has evolved (e.g. structured proteins can phase separate), but it still represents the most common method of collating testing and training datasets for predictor production. To test these algorithms with a “native” dataset, i.e., one similar to what they were trained and tested with initially, the “Standard” dataset was produced. Experimentally validated LLPS+ proteins were collected from an array of different LLPS+ databases via the meta-database MLOsMetaDB [56], and LLPS− proteins were identified as those with high-resolution x-ray structures from PDB. The datasets were then curated and culled as outlined in Fig. 1 to remove overlapping sequences.

**Fig. 2.**
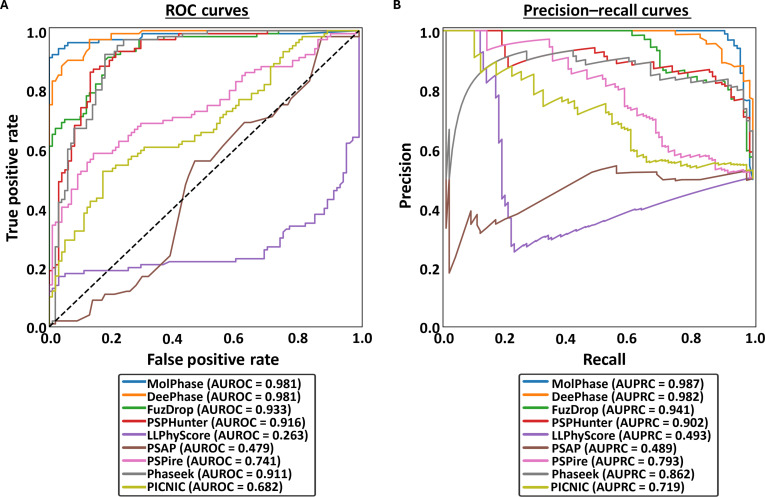
Analysis of liquid–liquid phase separation (LLPS) predictors using the “Standard” dataset. (A) Receiver operating characteristic (ROC) curves and (B) precision–recall curves (PRCs) of all 9 algorithms following prediction of the “Standard” protein dataset. Area under the ROC curve (AUROC) and area under the PRC (AUPRC) calculated for each. The black dashed line indicates the expected performance of a random classifier (AUROC = 0.5).

To assess the performance of each predictor, ROC curves and PRCs were constructed and the area under values were then calculated: AUROC and AUPRC, respectively. AUROC measures the probability that a randomly chosen LLPS+ protein is scored higher than a randomly chosen LLPS− protein, highlighting how well a model separates positives and negatives overall. AUPRC measures how precisely a model captures true positives across recall levels. Despite all predictors being trained and tested on similarly curated datasets, only 5 of the 9 algorithms possess AUROC scores with very high accuracy (>0.9) (Fig. 2A). Interestingly, the 2 structure-informed predictors included in this analysis had a good level of accuracy (PSPire: 0.741; PICNIC: 0.682) but were middling in comparison to the others assessed (Fig. S1a). This suggests that while structural features are key for the process of LLPS, sequence-derived features alone are sufficient to provide highly reliable LLPS propensity prediction.

Surprisingly, 2 predictors performed below random chance (0.5), actively placing proteins into the opposing categories: PSAP (0.479) and LLPhyScore (0.263) (Fig. 2A). LLPhyScore produces a linear weighted sum score of the top 8 contributing sequence factors as the final propensity score. The score considers whether these features are positively (e.g., disorder) or negatively (e.g., short-range electrostatics) correlated with a higher LLPS propensity. While this does allow for an easier interpretability of which features contribute to the overall score and to what extent, it also leads to vulnerability in the predictions. Proteins undergoing LLPS can have very diverse characteristics, contributing to the struggle to find the key features for identification and prediction. Therefore, a protein that has been experimentally shown to phase separate but has an “outlier” characteristic (e.g., low disorder or high electrostatics) could be incorrectly predicted by LLPhyScore. This appears to be less of an issue with other predictors, such as MolPhase and DeePhase, which utilize larger numbers of sequence properties and nonlinear machine/deep learning (e.g., random forest) to better understand the complex relationships between characteristics and handle “outlier” features. This does sacrifice some level of interpretability of each feature’s contribution but provides more accurate overall predictions. This also aligns well with the AUPRC score being much higher than the AUROC for LLPhyScore (Fig. 2A and B), suggesting that the proteins it is scoring correctly are only those with a high LLPS propensity.

PSAP scored much closer to the “random chance” boundary but was still not performing as well with this dataset. While it does utilize a large number of protein sequence features and a nonlinear random forest approach, issues likely come from the training. For this predictor, it was trained on only 90 high-confidence, experimentally identified PSPs. This is a relatively low number of proteins to use for training purposes as it can reduce the generalizability of the predictions, meaning proteins that do phase separate but have less “characteristic” features (e.g., lower disorder and no LCRs) are more likely to be incorrectly assessed.

### Disordered and folded proteins reveal structural bias in phase separation prediction

One of the issues with using a dataset like the “Standard” set for training and testing predictors is that it limits the diversity of the proteins included. Many proteins that are disordered have been shown to undergo phase separation [32]; however, not every disordered protein shows a ready propensity for this [32,77,78]. Additionally, a growing number of highly structured and folded proteins have been shown to undergo phase separation [79–82]. By using highly ordered protein sequences from the PDB as the main source of LLPS− proteins, the predictions made will therefore be biased toward ordered proteins being negative and disordered proteins being positive, which is not always correct. To assess if the predictors are impacted by this bias, the “Disordered vs. Folded” dataset was produced. As shown in Fig. 1, LLPS+ proteins were collated from MLOsMetaDB and categorized into disordered or folded utilizing Metapredict [59] (threshold of 0.5). LLPS− folded proteins were collected as usual from the PDB, but the disordered LLPS− proteins were taken from the DisProt [60] disordered protein database.

**Fig. 3.**
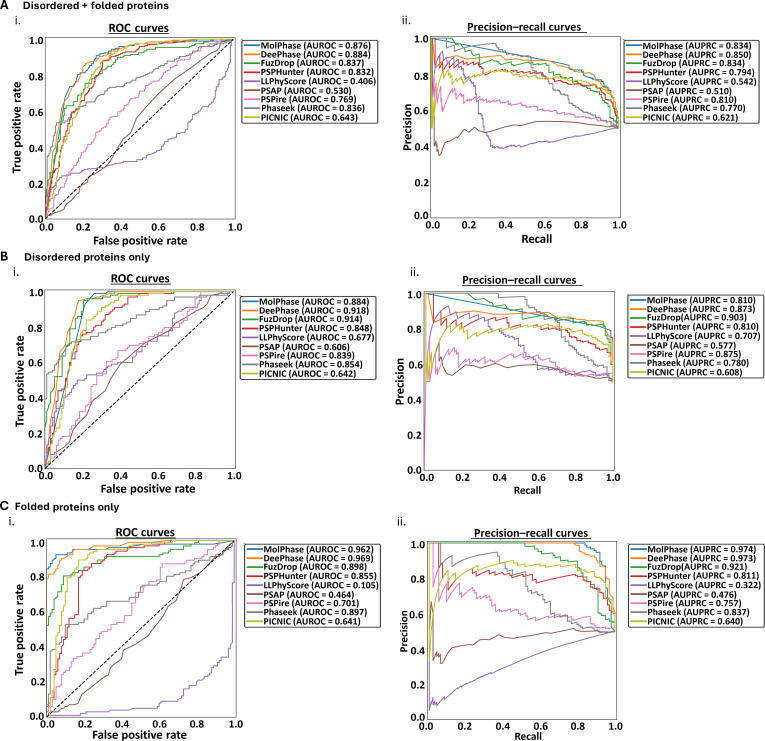
Liquid–liquid phase separation (LLPS) predictors vary in the ability to discriminate LLPS-positive (LLPS+) and LLPS-negative (LLPS−) proteins of different structural orders. The performance of each LLPS predictor was assessed on datasets consisting of equally mixed LLPS+ and LLPS− proteins. The datasets contained either (A) an equal mixture of disordered and folded proteins, (B) only disordered proteins, or (C) only folded/structured proteins. For each dataset, the (i) area under the receiver operating characteristic curve (AUROC) and (ii) area under the precision–recall curve (AUPRC) were calculated for each predictor. The black dashed line indicates the expected performance of a random classifier (AUROC = 0.5).

The mixed dataset (Fig. 3A) is similar in composition to the “Standard” dataset (Fig. 2) but is equally balanced with folded and disordered proteins of both LLPS+ and LLPS− classification. While the results initially seem similar to those of the “Standard” dataset, 6 of the 9 predictors showed a decrease in overall AUROC and AUPRC scores (Fig. 3A (i) and (ii)). This suggests a potential overfitting of the predictors to the idea that disordered means LLPS+ and folded means LLPS− as the more diverse the test set, the weaker the scores. Those that showed slight increases in AUROC and AUPRC (PSPire, PSAP, and LLPhyScore) still, however, remain middling to low in overall comparative performance (Fig. S1c (i)).

To further understand how these biases could be affecting the predictors, the different structure types were run individually through each algorithm. As expected, when assessing only disordered LLPS+ and LLPS− proteins, the same trend of decreased AUROC and AUPRC as with the mixed set is evident in the same 6 predictors (Fig. 3B (i) and (ii)). This solidifies the idea that many of these algorithms are overfitted, likely due to the overreliance on structured proteins for a negative dataset. Interestingly, one of the only algorithms showing an increase in AUROC and AUPRC in this dataset is PSPire, a structure-informed algorithm. PSPire was specifically designed to make assessments based on protein structure files while also not relying heavily on disorder for its predictions [26], something reflected by this dataset. The other 2 predictors showing an increase were the 2 lowest performers within the “Standard” dataset: LLPhyScore and PSAP. Both now score >0.6, well above the random chance line, which indicates that, unlike the other algorithms, these are not overfitted to the same idea of disorder.

However, the issues in LLPhyScore’s model becomes evident when considering only folded LLPS+ and LLPS− proteins (Fig. 3C). The AUROC score from the “Disordered-Only” dataset decreased by 0.572 when looking at folded proteins, suggesting that LLPhyScore is unable to discriminate the LLPS propensity of a well-structured protein, relying heavily on disorder attributes. In comparison to the standard dataset, all algorithms saw a decrease in AUROC and AUPRC scores, although most were only small changes. The same trend was observed even with moderate changes to the classification of disordered *vs.* folded via Metapredict, shown in Fig. S7 and Table S12.

To gain further understanding of the differences between LLPS+ and LLPS− disordered and folded proteins, GO analysis was undertaken on the “Disordered vs. Folded” dataset. Interestingly, numerous LLPS-associated molecular function GO terms [83,84] were enriched in both the LLPS+ and LLPS− disordered protein sets (Fig. S5) such as nucleotide binding (e.g., DNA binding, sequence-specific DNA binding, and messenger RNA binding) and protein kinase binding. Additionally, very few LLPS-associated molecular function GO terms were enriched in the LLPS+ folded protein dataset (Fig. S4). This suggests that some features currently thought of as LLPS associated and potentially considered by prediction algorithms are just disorder associated, further biasing results. In addition, some LLPS-associated features may be getting missed as they are enriched mainly in LLPS+ folded proteins and not disordered proteins, further highlighting the bias in current research toward the oversimplified idea that disorder equals LLPS+.

Overall, dissection of predictor performance based on the structural disorder of a protein has highlighted the common overfitting of predictors to the idea that the higher the level of disorder, the higher the propensity for LLPS (and vice versa) when this is becoming less of a scientifically accepted idea. Therefore, it is a necessity to develop more diverse training datasets to produce these predictors and aid in the avoidance of this disorder bias. Promisingly, however, the same 3 algorithms have formed the top performers across the “Standard” and “Disordered vs. Folded” datasets (Fig. S1a and c)—DeePhase, MolPhase, and FuzDrop. While being somewhat affected by bias in training, each of these predictors showed high levels of accuracy (AUROC and AUPRC > 0.8) no matter the dataset, suggesting these to be highly robust predictors for diverse proteins.

### Benchmarking predictors on a diverse and high-confidence dataset uncovers performance disparities

As previously discussed, a large confounding factor in the prediction of LLPS proteins is the lack of standardized benchmark datasets, especially for LLPS− proteins. The issues with this have been exemplified here with poorer prediction scores being returned depending on the composition and diversity of the datasets to be predicted. To tackle this, Pintado-Grima *et al.* [63] developed more strictly filtered LLPS+ and LLPS− sets of proteins from a wide variety of LLPS databases and protein structure sites, including DisProt for disordered LLPS− representation, the importance of which has been shown here. Part of this strict filtration includes the removal of any LLPS− proteins with documented interactions with any known PSPs or proteins related to LLPS. This should help reduce the inclusion of false negatives while retaining a higher level of structural diversity than standard datasets.

While the effects of using this “Benchmark” dataset for training a predictor will not be tested here, utilizing it as a testing dataset can give some insight into the suitability of the current training of these predictors. For example, when compared to the results from the “Standard” dataset, if the AUROC score increases, it suggests that the algorithm performs well and was being “dragged down” by incorrectly labeled proteins, most likely false negatives (e.g., LLPS+ folded proteins wrongly described as LLPS−). In the opposite case, with a decrease in AUROC, it suggests that the predictors may have learned incorrect correlations or artifacts from being trained on “noisy” data containing false positives and negatives.

**Fig. 4.**
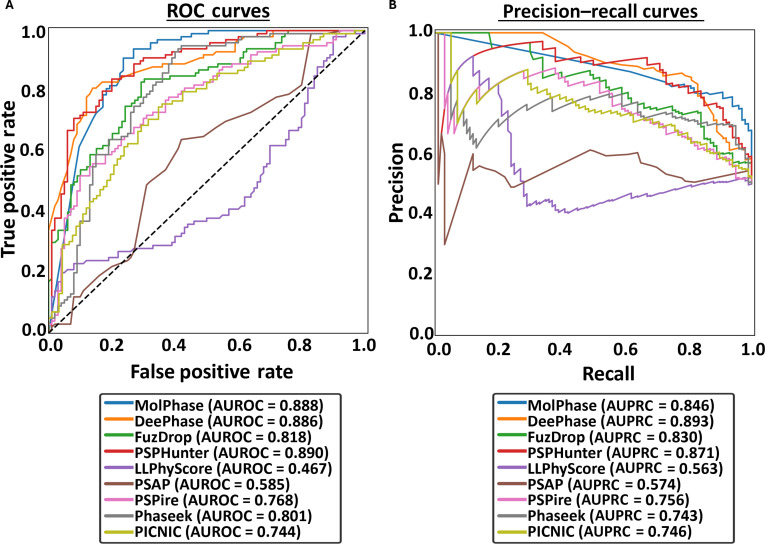
New “Benchmark” datasets highlight the issues with current testing and training datasets. (A) Receiver operating characteristic (ROC) curves and (B) precision–recall curves (PRCs) of all 9 algorithms following the prediction of the “Benchmark” protein dataset. Area under the ROC curve (AUROC) and area under the PRC (AUPRC) calculated for each. The black dashed line indicates the expected performance of a random classifier (AUROC = 0.5).

The top 4 performers from the previous datasets (MolPhase, DeePhase, FuzDrop, and Phaseek) all show an ~0.1 decrease in AUROC compared to the “Standard” dataset (Fig. 4A), suggesting impacts of “noisy” training data. MolPhase, DeePhase, and Phaseek all employ only PDB sequences as the negative dataset, likely accounting for these issues. Interestingly, with DeePhase, there is a large difference between AUROC and AUPRC (Fig. 4B). While AUROC decreased, AUPRC stayed near “Standard” dataset levels, suggesting that DeePhase still performs well with identifying true positives, and thus, the issues are likely with the prediction of the negative class. FuzDrop, on the other hand, employs the human proteome (minus known PSPs) as its negative dataset. While this does improve upon the PDB-based datasets in terms of structural and protein diversity, it also very likely contains many false negatives that are yet to be identified, contributing to noise.

LLPhyScore, PSAP, and PICNIC all showed the opposite trend, with increases in AUROC from the standard dataset. While suggesting that these predictors are being affected by incorrect labeling, their overall ranks in comparison to others remain the lowest (Fig. S1b).

Overall, this exemplifies the importance of producing standardized benchmark datasets for both the training and testing of predictors. The currently used PDB and human proteome negative sets have inherent issues in their lack of protein diversity and likely inclusion of false negatives, respectively. The inclusion of disordered negative proteins (e.g., DisProt) will aid in the removal of bias by increasing dataset diversity but will likely introduce false negatives similar to those seen with the human proteome. Therefore, the approach taken by Pintado-Grima *et al.* [63] utilizing a diverse negative dataset with stringent filtration provides the best balance of diversity with reducing type II errors.

### Mutation-level analysis exposes weaknesses in predictor robustness and adaptability

A growing area of interest in LLPS research is the contribution of single residues or small regions in the protein sequence in driving the phase separation process. Numerous PSPs have been identified as being driven by such regions, and when these get mutated or removed, it severely inhibits or completely ablates the ability for those proteins to undergo LLPS [64–66]. As aberrant phase separation has been linked to a variety of diseases [7–11], it is of interest to be able to predict the following: (a) regions thought to drive phase separation and (b) the impact of mutating these regions on overall protein phase separation ability. While a growing number of algorithms are providing residue-level information involved in predictions (see Table 1), it is often stated that current predictors are unable to detect these changes reliably.

To investigate the ability of current second- and third-generation phase separation predictors to interpret the impact on LLPS caused by such changes in protein sequence, the “Mutations” dataset was produced. Alongside categorizing LLPS+ proteins, LLPSDB v2.0 [67] also categorizes experimentally established mutations in PSPs that can alter or completely disrupt its ability to undergo LLPS. The WT sequences for these mutations were collected, and any with >40% similarity to those used in the predictor training datasets were removed. This returned a set of 12 proteins with 24 unique mutations sequences. For the purpose of this analysis, WT sequences represent the LLPS+ class, and the mutant sequences represent the LLPS− class.

As the class sizes for this dataset are notably lower than those of the other testing datasets used, the AUROC and AUPRC must be interpreted carefully. With such a small dataset, these analysis parameters are particularly susceptible to even single incorrect predictions, causing large swings in the results. Additionally, the results will have a limited resolution due to fewer datapoints causing coarse and less interpretable curves. Despite this, these metrics still provide good indicative insights into the predictors, and 95% CIs have been provided to aid in careful interpretation (Fig. S1d).

**Fig. 5.**
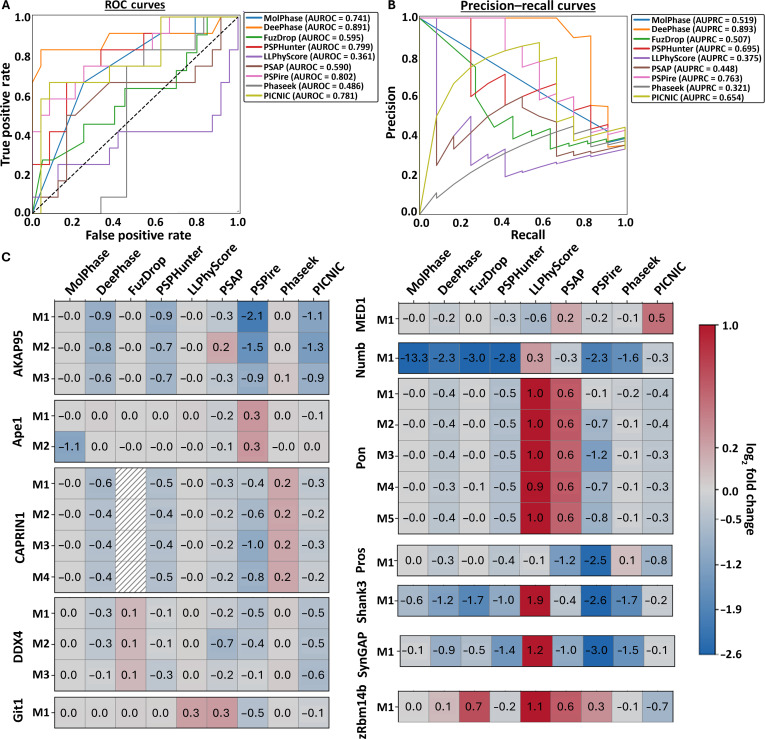
DeePhase, PSPHunter, PSPire, and PICNIC are able to consistently detect changes in liquid–liquid phase separation (LLPS) propensity induced by small amino acid changes. (A) Receiver operating characteristic (ROC) curves and (B) precision–recall curves (PRCs) of all 9 algorithms following the prediction of the “Mutations” protein dataset. Area under the ROC curve (AUROC) and area under the PRC (AUPRC) calculated for each. The black dashed line indicates the expected performance of a random classifier (AUROC = 0.5). (C) log_2_ fold change calculated of prediction scores from the wild-type (WT) LLPS-positive (LLPS+) sequence to the mutant LLPS-negative (LLPS−) variants. Red colors represent an increase in mutant LLPS propensity compared to WT. Blue colors represent a decrease in mutant LLPS propensity compared to WT.

Across all predictors, the AUPRC scores (Fig. 5B) have reduced compared to those of the “Standard” dataset, with 3 predictors having AUPRC below 0.5 (LLPhyScore, PSAP, and Phaseek) and only 1 predictor being above 0.7 (DeePhase, AUPRC 0.893). In the context of this dataset, the LLPS+ class is 33% of the dataset, meaning a random classifier would be expected to have an AUPRC of ~0.33, a threshold of which all algorithms exceed except Phaseek, which is just slightly under (0.321). This overall decrease and low levels of AUPRC show that the precision and recall in this situation is reduced, indicating that all predictors (except DeePhase) struggle with correctly identifying the positives from the negatives.

Looking at the AUROC (Fig. 5A) shows us that top predictors have changed drastically. DeePhase remains the top performer in both metrics, showing minimal decreases in performance across all datasets assessed so far. However, the other 2 predictors making up the top 3 include PSPire and PSPHunter. As discussed previously, PSPire is a structure-informed predictor that has performed well in terms of AUROC but middling in comparative analysis for the other datasets. Alongside the other structure-informed predictor PICNIC, they have climbed the ranks when assessing the effects of mutations, suggesting that including structural information in predictions may not have a large impact on the overall prediction score but bolsters the ability to identify impacts on LLPS propensity caused by small mutations. The other top performer, PSPHunter, is an interesting algorithm as it has been designed with this specific purpose in mind—identifying the phase-driving regions of proteins and the impacts of single mutations on propensity. For this study, the protein-level prediction module was used to enable calculation of analysis metrics and direct comparison to other algorithms, rather than the other purpose-built modules. Even in this instance, PSPHunter scored well, providing a high level of accurate ranking.

While it may be difficult for the predictors to directly give a binary “yes or no” answer based on these small changes, they may be able to represent the change in LLPS ability as a decrease in propensity. For example, DeePhase identifies proteins with a score ≥0.5 as LLPS+ and those with <0.5 as LLPS−, and as such, we would expect the WT sequence to be ≥0.5 and the mutant sequence to be <0.5. However, as detecting such small changes is a difficult classification task, the differences may be more in the lines of 0.85 for WT and 0.65 for the mutant. To assess this, the difference in LLPS prediction score between WT and mutant sequences was calculated, shown in Fig. 5C. Here, we can see 3 main outcomes for the predictors. The first is exemplified strongly by LLPhyScore and partially by PSAP in which an increase in LLPS propensity is often seen for the LLPS− mutants. This likely links to issues identified previously with these predictors in that they appear to be affected by bias and a lack of diversity in their training, and as such, they have learned incorrect patterns in sequence features *vs.* LLPS propensity. The second type is shown well by MolPhase and Phaseek, which are unable to detect changes caused by the LLPS− mutations compared to the WT sequence. For these predictors, residue-level changes have limited impact on their overall score, and as such, they are better suited for protein-level investigations rather than residue-level ones. The final type is represented by PSPire and PSPHunter in which quite large decreases in score are often reported. PSPHunter is the only algorithm that detected a decrease in almost all mutations and never reported an increase, showing that its built-for-purpose algorithm is working optimally.

Overall, only a small subset of predictors were able to accurately detect decreases in LLPS propensity caused by small mutations and, even then, are better represented as a fold change from WT rather than a binary result. PSPHunter is a built-for-purpose algorithm providing highly accurate predictions for small mutations in its protein-level predictions with additional modules allowing for further investigation at the region and residue levels. Interestingly, structure-informed predictors also excel in this area compared to their performance with other datasets, suggesting that structural information may aid in the identification of small regions and residues rather than a protein-level overall prediction. Interestingly, there appears to be little crossover in the key features of the top-performing predictors that are unique from those with weaker performance. Both sequence-based predictors, DeePhase and PSPHunter, do employ word2vec embeddings, treating residues and domains as “words” in vectors, which have been used for many types of protein prediction [85–88], potentially explaining their success. A more in-depth analysis is beyond the scope of this study but could provide key insights into which machine learning or protein-level features are unique to accurate mutation effect prediction and if structure could play a part in improving it further.

### Reduced accuracy on viral proteins suggests a distinct molecular signature not captured by current models

An area of LLPS research that has been recently increasing in popularity is that of LLPS in viral replication cycles. Many viruses form replication centers in the cell by large-scale remodeling of cellular membranous organelles such as the Golgi apparatus [89], endoplasmic reticulum [90–93], and lysosomes [94,95]. This allows for the formation of a “viral factory” in which key cellular and virus components are concentrated to allow efficient replication and virion production [96]. However, recently, an increasing number of viruses have been shown to induce the formation of liquid organelles in order to build their viral factories, driven by viral proteins via the process of LLPS [97–102]. This has had a large impact on the understanding of virus replication cycles and how viruses are able to hijack cells successfully, as well as opening avenues for potential new approaches to antiviral treatment [103,104]. It is therefore important to be able to accurately and rapidly screen viral proteins predicted to phase separate to accelerate research into virus-based LLPS. However, viral proteins are underrepresented in many databases used to collate training datasets [43] and often do not have homologs in other proteomes [44]; thus, predictors are unlikely to have learned any virus-specific patterns.

To investigate how well predictors can handle viral proteins, a “Virus” dataset was produced. The LLPS+ class was collected from BAV-LLPS-DB, a newly produced LLPS database dedicated to PSPs from bacteria, archaea, and viruses [68]. For the curation of the LLPS− dataset, the “Standard” approach was taken in which virus-specific proteins with high-resolution x-ray structures were chosen. While the issues with using PDB-only LLPS− sequences have been discussed thoroughly here, it was chosen for the “Virus” dataset for 2 main reasons. First, it ensures that the dataset is as similar to the “Standard” one as possible, providing the clearest comparison to determine if the predictors face issues with virus-specific sequences rather than a mixed set. Second, due to the limited number of entries in the LLPS+ class and the need to condense the LLPS− dataset to match, the PDB dataset currently provides the highest likelihood of containing fewer false negative proteins as the currently limited understanding of LLPS within viral proteins makes it difficult to benchmark.

**Fig. 6.**
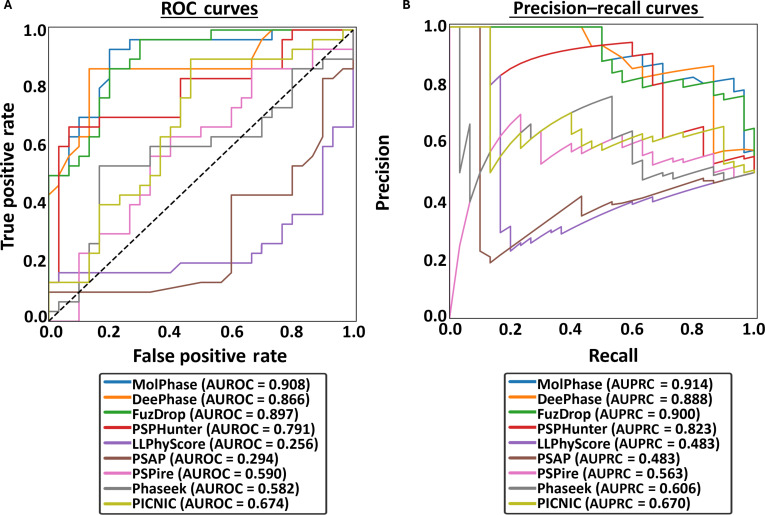
Liquid–liquid phase separation (LLPS) predictors are less confident assessing virus-specific proteins. (A) Receiver operating characteristic (ROC) curves and (B) precision–recall curves (PRCs) of all 9 algorithms following the prediction of the “Virus” protein dataset. Area under the ROC curve (AUROC) and area under the PRC (AUPRC) calculated for each. The black dashed line indicates the expected performance of a random classifier (AUROC = 0.5).

Across all predictors tested, the AUROC and AUPRC of each (Fig. 6A and B) decreased compared to that of the “Standard” dataset (Fig. 2A and B). The largest reduction was with Phaseek, with the AUROC decreasing by 0.329 from 0.911 to 0.582. DeePhase, while still performing well (0.866), dropped in the rankings (Fig. S1e), with a decrease in AUROC score of 0.115. Despite this, the top 3 algorithms still remained as MolPhase, DeePhase, and FuzDrop, showing their high accuracy across all datasets, except for the “Mutations” dataset.

To assess what may be contributing to the differences in score, protein feature analysis of the datasets used in this study was undertaken (Fig. S6). All features analyzed showed significant differences between the LLPS+ and LLPS− classes except the NCPR (Fig. S6b). Interestingly, within the LLPS+ classes, viral proteins show significantly different presentations than the “Standard” and “Benchmark” proteins in 3 of the assessed features. A common feature of LLPS+ proteins is the presence of LCRs [105]; however, the LLPS+ viral proteins showed a significantly lower fraction of low-complexity residues than found in the “Standard” and “Benchmark” datasets. A similar trend could also be seen with protein length, with LLPS+ viral proteins having significantly shorter sequences than other LLPS+ proteins, a trend that has been previously noted when comparing viral proteins to matched metazoan proteins [106]. In contrast, however, LLPS+ “Virus” proteins show a significantly higher hydrophobicity than other LLPS+ proteins, similar to the levels of LLPS− proteins, with LLPS− “Virus” proteins being even higher. Lower hydrophobicity is generally associated with a higher LLPS propensity [45,46,52], highlighting potential reasons why predictor performance on the “Virus” dataset was lower.

Overall, this highlights that viral proteins likely have a somewhat different “molecular language” from that of proteins from different organisms. Thus, the training of these predictors, often either on a virus-underrepresenting database or on the human proteome alone, does not capture virus LLPS as accurately. To further investigate this, a dataset should be created of virus-only proteins and utilized to train high-performing prediction algorithms either alone or alongside standard datasets to understand if these patterns can be used to ascertain more accurate prediction scores. This could then be similarly applied to other underrepresented proteins to create an array of high-accuracy specialized predictors.

### Future perspectives for LLPS protein sequence prediction

Following analysis of these 9 LLPS predictors on diverse sets of proteins, numerous points of debate have been raised regarding LLPS predictor design and usage going forward. The assessment of predictor performance across protein datasets of varied structural classification highlights a clear bias of both algorithm training and performance toward disordered proteins being LLPS+ and negative proteins being LLPS−. Research into LLPS systems is moving away from this idea with growing evidence that not all disordered proteins undergo an LLPS transition [35,77,78] as well as an increasing number of folded proteins being implicated in LLPS activities [79–82]. It is therefore imperative that LLPS predictor research develops alongside this, moving away from the use of PDB-derived folded structures as “negatives” and toward collation of more diverse training and testing datasets. This could take the form of standardized benchmark datasets, such as the approach taken by Pintado-Grima *et al.* [63], or via the adoption of more rigorous dataset curation practices to represent the true variety of both LLPS+ and LLPS− proteins. Additionally, testing of these algorithms, whether by the developers or by an independent party, should also utilize diverse protein datasets to enable clearer interpretation of the predictor’s usage across numerous scenarios.

An interesting aspect of this comparative analysis involved assessing the potential “third generation” of LLPS prediction models that incorporate structure-informed features in their predictions [26,31]. While the 2 tested here performed consistently in the mid-range across all algorithms, they showed increased performance when assessing the impact of single-amino-acid or short-region mutations known to abolish LLPS ability (Fig. 5). This suggests that structure incorporation can aid in the residue-level resolution of predictions, an aspect that many predictors currently struggle with. While the incorporation of these features into LLPS predictors is still in its infancy, further development and refinement of this approach could provide a very interesting future for LLPS prediction.

**Fig. 7.**
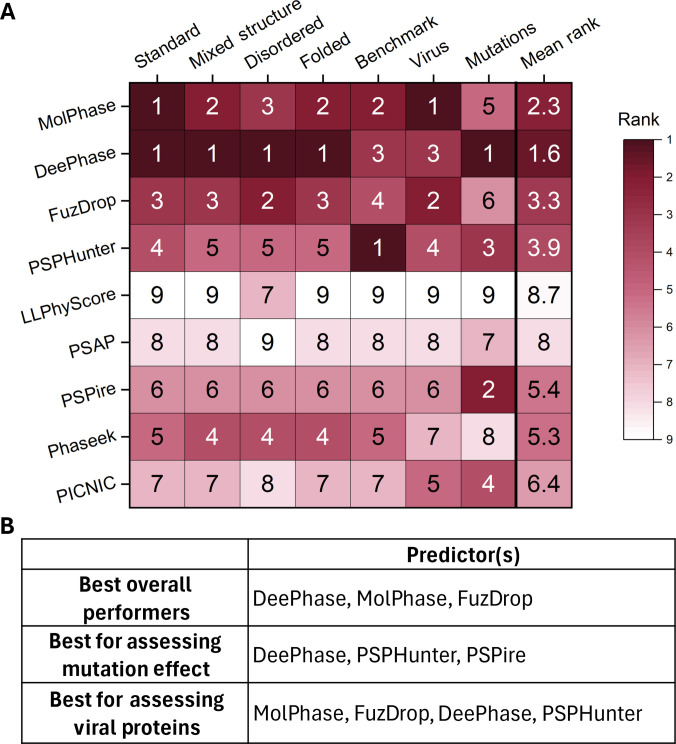
Overall performance of liquid–liquid phase separation (LLPS) predictors. (A) Heatmap showing the “rank” of all 9 algorithm across each dataset based on the highest (first) to lowest (ninth) AUROC scores. The overall rank was determined by collecting the mean value for each predictor. Dark red colors represent better-performing predictors, and light red to white represent the worse-performing predictors. (B) Table showing the best-performing algorithms for specific usage situations.

To compare the model architecture and prediction approach of top-performing prediction algorithms, the overall performance and performance on individual datasets was assessed, shown in Fig. 7. The top 3 performers, DeePhase, MolPhase, and FuzDrop, all utilize different model constructions (Table 1), and while a full analysis of model construction would be beneficial to determine a potential optimal build, this is beyond the scope of the study. However, from the key features analyzed, it can be noted that the top 3 general performers uniquely consider Shannon entropy, a protein feature that has been implicated in LLPS, being in careful balance with enthalpy [25]. While unlikely to be the only feature separating prediction performance, it will be an important feature to consider for future algorithms.

One of the biggest takeaways from the comparative analysis and assessment of performance (Fig. 7) is the large impact that the context of proteins being assessed or the information being gathered has. Performance could differ greatly for the same algorithm depending on the class of proteins being assessed, such as Phaseek consistently ranking fourth for all “Disordered vs. Folded” datasets but seventh when assessing viral proteins (Fig. 7). Additionally, many algorithms faltered in the context of predicting the impact of LLPS-abolishing mutations on LLPS+ proteins. As small mutations have been linked to aberrant LLPS and disease outcomes [7,64–66], this is an important experimental context to be able to accurately predict. Therefore, these issues suggest that a beneficial direction forward for the field of LLPS prediction may not be the goal of one gold-standard algorithm to predict all protein types and contexts but instead a suite of purpose-built models. This could include algorithms specifically trained for underrepresented proteins that may have their own unique signatures (such as viral proteins), those designed for detecting residue-level contributions and the impacts of mutations of these residues, and potentially those designed to detect folded LLPS+ protein signatures and an alternative for disordered LLPS+ protein signatures.

## Conclusion

As the research area of LLPS in biological systems continues to grow and merge with other disciplines, the need for accurate and user-friendly LLPS predictors gets larger. This has led to the development of a wide array of second-generation machine learning algorithms designed to learn the “molecular language” of proteins and identify features characteristic of PSPs. However, with the variety of predictors available, each utilizing different protein characteristics from sequences or structures and employing various machine or deep learning approaches, it is difficult to decide on which to use. This study provides an unbiased comparative analysis of 9 of the currently available second- and third-generation predictors across a comprehensive collection of datasets.

DeePhase provided the best overall performance in all datasets, never falling below the top 3 performers (Fig. 7A), indicating that it provides accurate predictions across a diverse range of proteins. MolPhase also performs similarly to DeePhase across the board, only faltering when assessing the impact of small mutations on protein-level LLPS propensity. MolPhase does, however, provide a larger amount of insight into residue-level details for each metric considered, as outlined in Table 1. FuzDrop is the third best-ranked predictor; however, it is outperformed on every dataset by another, although it does beneficially provide information on specific regions in the protein that are predicted to promote LLPS or aggregation. Therefore, for the purposes of screening proteins for LLPS propensity, DeePhase or MolPhase provides the most accurate results.

As many researchers are interested in the investigation of residues or regions that are key for driving LLPS and such can be mutated to abolish or disrupt it, it is important to identify predictors useful in this specific situation. Somewhat surprisingly, DeePhase was the best performing in this dataset in terms of AUROC despite not being specifically designed for this purpose. Even when considering the evaluation metric of fold change rather than a binary “yes or no”, DeePhase was able to report a decrease in most of the mutant sequences compared to WT. However, here it was outperformed by PSPHunter, the built-for-purpose predictor for identifying deleterious mutations and phase-driving regions. Interestingly, 2 other high performers here were the 2 third-generation structure-informed predictors PSPire and PICNIC, which had otherwise performed middling in the other datasets. This suggests that while structural data may not be as important as sequence data for prediction of an overall protein score, it can provide good insight into the effect of small mutations on overall propensity. Therefore, to investigate the impact of small mutations on a protein and their phase-driving regions, a mixed-approach utilizing multiple predictors will likely give the most comprehensive insight, specifically DeePhase, PSPHunter, and PSPire.

Another area of growing research regarding LLPS is the involvement of it in viral replication processes [107,108]. LLPS is being implicated as a key process in numerous viruses, and as such, the need to be able to predict which viral proteins are likely to undergo this is increasing. However, many of these algorithms are trained on datasets in which viral proteins are underrepresented and so any LLPS-inducing patterns unique to viral proteins are likely being missed. This study has identified that this is a likely bias in many of the predictors currently existing, which may be due to different sequence features of molecular language present in viral proteins. Despite these issues, the best performer for these sequences was MolPhase, which showed limited AUROC differences in comparison.

Throughout this study, the issues that currently persist in the training and testing datasets used for many predictors have been well-documented. They are currently showing bias toward the outdated view that disorder guarantees LLPS and majority-folded proteins cannot undergo LLPS. It is imperative to create both LLPS+ and LLPS− datasets that represent the wide variety of proteins in each class. One method of doing this in this study is shown by Pintado-Grima *et al.* [63], in which proteins from well-structured and disordered protein databases were used in the LLPS− dataset and were strictly filtered to remove as many false negatives as possible. The use of such standardized benchmark datasets could then lead to a new generation of LLPS predictors with the best prediction accuracy yet.

## Data Availability

All data generated and analyzed during this study are included in this published article and its Supplementary Materials files and available at the publicly available GitHub repository: https://github.com/BasicallyKate/LLPS_Benchmarking.
